# Long-term monitoring of intracranial pressure in freely-moving rats; impact of different physiological states

**DOI:** 10.1186/s12987-020-00199-z

**Published:** 2020-06-09

**Authors:** Sajedeh Eftekhari, Connar Stanley James Westgate, Katrine Printz Johansen, Signe Rath Bruun, Rigmor H. Jensen

**Affiliations:** Danish Headache Center, Department of Neurology, Glostrup Research Institute, Rigshospitalet-Glostrup, University of Copenhagen, Nordstjernevej 42, 2600 Glostrup, Denmark

**Keywords:** Intracranial pressure, Neurophysiology, Waveforms, Telemetry system, Freely-moving, Anesthesia, Cerebrospinal fluid, Choroid plexus, GFAP

## Abstract

**Background:**

Elevated intracranial pressure (ICP) is observed in association with a range of brain disorders. There is limited insight into the regulatory mechanisms of ICP under physiological conditions, and consequently also under pathological conditions. Thereby, to understand the mechanisms underlying ICP dynamics, precise, valid and long-term ICP recordings are of importance in the preclinical setting. Herein, we used a novel telemetric system for ICP recordings which allowed for long-term recordings in freely-moving rats. The aim was to investigate ICP dynamics under different physiological states and investigate how factors such as surgery/recovery, body position, light–dark, co-housing, weight and anesthesia may influence ICP and its waveforms.

**Methods:**

A telemetric device was implanted epidurally in rats and signals were recorded continuously for up to 50 days (n = 14). Recording was divided into three experimental periods: a surgical recovery period (RP), a physiological period (PP) and an experimental period (EP). Histology was performed to study the morphology of implanted rats and non-implanted rats (n = 17).

**Results:**

For the first time, we can demonstrate continuous ICP recordings in freely-moving and co-housed rats for up to 50 days with a high degree of stability. The mean ICP in the recording periods were; RP: 3.2 ± 0.6 mmHg, PP: 5.0 ± 0.6 mmHg and EP: 4.7 ± 0.6 mmHg. In the RP, the ICP was significantly lower compared to the PP (P = 0.0034). Significant light–dark difference in ICP with 21% increase in respiratory slow-wave amplitude was observed in the co-housed animals but not in single-housed animals. The ICP signal was raised during the dark period relative to the light (Δ0.3 ± 0.07 mmHg, P = 0.0043). Administration of anesthesia gave a short-term increase in ICP followed by a significant decrease in ICP. No signs of tissue damage or inflammation were found in the implanted brains.

**Conclusions:**

ICP dynamics were influenced by several factors such as, use of anesthesia, light–dark difference and housing conditions. Our study demonstrates the importance of performing ICP physiological measurements in freely-moving animals. This has significant implications for moving the preclinical research field forward in order to properly study ICP physiology during disease development and to explore drug targets for alleviating increased ICP.

## Introduction

The regulation of intracranial pressure (ICP) is fundamental in providing a stable environment to enable normal brain function. Due to the finite volume capacity inside the skull, ICP is determined by the three components occupying the intracranial space; the cerebrospinal fluid (CSF), the blood and the brain tissue, as postulated by the Monro-Kellie hypothesis [1]. ICP elevation is observed in a range of cerebral pathologies, such as traumatic brain injury (TBI), ischemic stroke, hydrocephalus and idiopathic intracranial pressure (IIH). These disorders are among the most disabling and cost-intensive brain disorders with a high economic burden to society [2]. ICP is frequently measured in the clinic for diagnostic purposes, but yet there are no precise and non-invasive ICP recordings. Common techniques for ICP monitoring in clinical practice are intraventricular, parenchymal measurements or lumbar puncture (LP) measurements. However, these methods only provide a short-term pressure reading during these procedures or it is limited to few days, due to a progressively increasing risk of infection and patient mobility [3, 4]. Furthermore, our knowledge of the normal regulation of ICP is very limited as ICP monitoring in healthy subjects is ethically unacceptable. For this reason, there are no true reference values for ICP in healthy humans. Existing data which are considered “normal” ICP values are derived from examinations performed in patients needing ICP monitoring due to suspected disturbance in ICP or taken from measurements of lumbar CSF pressure. This demonstrates a great necessity for consistent and precise ICP monitoring in animal models, where ICP can be measured across the day and over longer periods of time in order to better understand the regulation of ICP.

ICP measurements in the preclinical setting are also of great importance to investigate how the dynamics and mean level of ICP may change under normal physiological conditions as well as during disease development. Furthermore, ICP measurements in animals are useful to investigate potential new therapeutic strategies. Refined equipment specifically designed to measure ICP in rodents has been lacking, and as such it has been difficult to accurately measure ICP. Previous efforts have investigated different methods and locations for ICP monitoring [5]. However, only short-term monitoring has been possible because some of these measurement sites are highly invasive as well as yield severe complications such as infections and degeneration of the cerebral tissue. Additionally, the use of anesthesia or sedation prior to ICP monitoring is required in most of these methods. A consequence of sedating the rat prior to ICP measurements is that the ICP data are only obtained in a resting position, single-housing and under a possible influence of anesthesia [6–8]. Thus, these methods are not well suited for long-term, continuous recording. Previously, we performed long-term monitoring of ICP in rats from epidural space using a fluid-filled catheter [8]. Although this method was a major improvement for measuring ICP, there were several limitations; the transducer is not permanently implanted, which prevents the possibility of continuous ICP recordings over larger time intervals and therefore the ICP recordings were only performed on selected days for up to six sessions. Further, this ICP method still depends on repeated use of anesthesia/sedation before each recording to immobilize the animals with no possibility to monitor ICP while they are freely moving or co-housed. Importantly, the fluid-filled systems can develop air bubbles in the system, resulting in erroneous ICP values.

Recently, fully implantable telemetric probes with specific ICP transducers have been developed to measure ICP continuously in freely-moving rats. This telemetry system allows long-term recordings under conditions unhindered by anesthesia or restraint [9]. This system also has a strong translational perspective as telemetric ICP monitoring has recently been established for clinical applications in small case series [10–12]. So far, only a few studies have used this preclinical system, one in male rats with ICP monitoring for up to 28 days and two in a TBI model with ICP monitored for 5–6 days [9, 13, 14]. However, a long-term validation of the system is lacking to date. Further, all previous studies have been performed in single-housed animals. Therefore, we aimed to investigate the feasibility of long-term and continuous ICP recordings from the epidural space in freely-moving rats with the possibility to be co-housed. Further, since there is limited data on ICP dynamics under normal physiological states, even in rodents, we aimed to investigate the ICP dynamics during different physiological conditions. The current study explored the impact of surgery/recovery, body positions, normal weight gain, light–dark variations and housing conditions on ICP. Since most previously used ICP monitoring methods are dependent on the use of anesthesia/sedation prior to ICP measurements, we also evaluated effect of anesthesia on ICP. Detailed histological examination was performed to ensure that implanted brains were intact. This knowledge is of great importance to increase our understanding of the basic mechanisms of ICP regulation and to develop new treatments for elevated ICP.

## Materials and methods

### Animals and groups

A total number of 17 female Sprague–Dawley (SD) rats (Taconic, Denmark) were used (weight: 240–310 g, age: 12–15 weeks at the time of the experiment). Rats were housed in the animal facility at Research Institute, Rigshospitalet-Glostrup, Denmark, and allowed to acclimatize for at least 1 week after arrival. The experiment was approved by the Danish Animal Experiments Inspectorate (2014-15-0201-00256). The rats were kept at a constant temperature of 22 ± 2 °C, 12-h reversed light/dark cycle (dark from 9.30 a.m. to 9.30 p.m.) with ad libitum access to food and water. The study was divided into two sub studies: one evaluated the feasibility of implantation of the telemetry system and ICP monitoring for 15 days, referred to as short-term ICP monitoring (n = 4). In the second study, continuous long-term ICP monitoring was performed for up to 50 days, referred to as long-term monitoring (n = 10). Non-implanted rats served as controls for the histology work (n = 3).

### ICP telemeter implantation and ICP recording

**Fig. 1:**
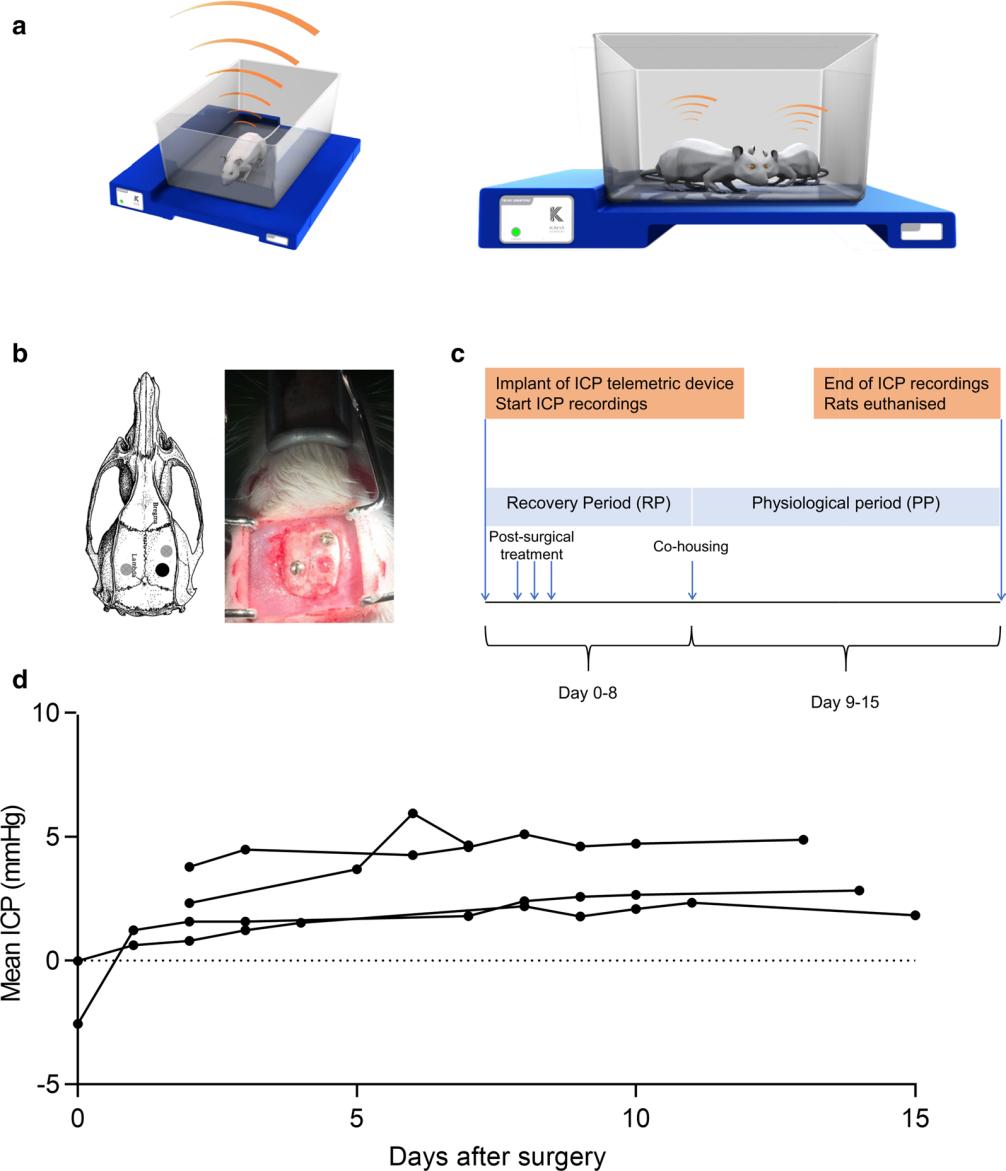
Telemetric system and implantation of telemeter: **a** ICP was measured by Kaha Sciences rat telemetry system (Kaha Sciences Ltd, Auckland, New Zealand). The system consists of an ICP telemeter for pressure measurement which is implanted in the animal. Implanted animals are then housed in their home cage placed on a SmartPad (in blue) with the possibility to co-house two implanted animals. The SmartPad functions as a power supply and wireless link to the telemeter for wireless data transmission. This system enables digital data transmission, wireless inductive power supply and 24/7 digital data collection from telemeters implanted in freely-moving, conscious animals living in their home cages. The ICP signal obtained is relative to the room pressure at that time. Images are reproduced with permission from Kaha Sciences. **b** Schematic illustration of the epidural placement of the sensor tip (black) and two anchoring screws (grey) localized in relation to Bregma. The image to the right obtained from the surgical procedure showing the beveled drill/thinned hole for the sensor tip and the two screws. The drill hole for the epidural sensor tip is made without penetrating dura mater. **c** Study design of the short-term study with a recovery period (RP) followed by a physiological period (PP). **d** Non-continuous individual daily mean ICP values for each of the rats included in the short-term ICP monitoring study (*n *= 4). The ICP measurements were obtained in a daily time-window of 2–5 h from day 0 or day 2 post-surgery and continued for up to day 15. Each dot represents the mean ICP value of each rat on the given day. The dotted line represents zero pressure

ICP was measured by the Kaha Sciences telemetry system (Kaha Sciences Ltd, Auckland, New Zealand, Fig. 1a). The system consists of a fully implantable telemetric device with a telemetric body and a sensor-tipped catheter. Animals were anesthetized with 2.7 ml/kg hypnorm/midazolam by subcutaneous (s.c.) injection. Antibiotic (10 mg/kg, Baytril, enrofloxacin, Bayer) was given s.c. prior to the surgery. Body temperature was maintained at 37 °C throughout the surgical procedure. A midline incision was made in the lower part of the abdomen and the telemeter body was placed in the abdominal cavity and sutured to the abdominal wall muscle. The muscle layer was sutured leaving the catheter outside the suture line. The catheter with its sensor tip was then tunneled through the subcutaneous space with a trocar to the base of the skull. The abdominal skin incision was closed with suture clips. Subsequently, the rat was placed in a stereotaxic frame to stabilize the head for placement of the ICP catheter and a dorsal midline incision of the scalp was made. The skin was removed to expose the bregma. For the placement of the sensor tip, a hole was drilled and thinned to transparency using a dental drill in the bone at least 7 mm posterior from bregma without penetrating the dura mater. The dura mater was exposed and bone residues were removed carefully to avoid penetration or damage. Two smaller holes on both sides of the sensor tip hole were made to fit anchoring screws. Two screws were screwed into these holes without penetrating through the brain surface. The ICP sensor tip was then inserted into the sensor tip hole to lie epidurally (between the skull and dura mater) (Fig. 1b). Gel Foam (Pfizer, Germany) was gently inserted at the catheter insertion point with a mesh patch placed over and tissue adhesive (Histoacryl, B.Braun, Germany) was used to secure the patch to the skull. Dental impression material (Coltene President, Switzerland) was applied followed by suturing the scalp incision. After surgery, the rats were placed in their home cages upon a Smartpad and ICP recording started (Fig. 1a). For data acquisition from Smartpad, recording and display, PowerLab and LabChart software (v8.0, ADInstruments,) were used. The telemeter sampled ICP at 2 kHz, where the Smartpad lowpass filter at 1 kHz gave a final sampling frequency of 1 kHz.

#### Post-surgery treatment and animal welfare

After surgery and the two following days the rats received 2–4 ml saline, 0.03 mg/kg buprenorphine (Temgesic, Pharmaceuticals limited, UK), 5 mg/kg carprofen (Rimadyl, Pfizer), 10 mg/kg enrofloxacin (Baytril, Bayer) all given s.c. and 0.4 mg/kg buprenorphine mixed in Nutella (Ferrero, Germany). In order to obtain proper and valid ICP data it is of great importance that the animals recover well from surgery and remain healthy, therefore body weight and water intake was monitored daily for a week post-surgery. Thereafter weight was monitored once a week. The following days after surgery, the rats lost some weight (< 15%) and from day 7 weight and water intake increased (Additional file 1: Figure S1). It was observed that their general well-being and activity level also increased.

#### Initial resting ICP and body position

After surgery (day 0), the proper placement of the sensor tip and the response of the ICP signal were confirmed by compressing the jugular vein giving an increase in ICP. It is known that ICP changes in humans with different body postures [15, 16]. In order to investigate if this also happens in rats and to confirm the ICP signal, postural changes were checked by holding the rat with its head up (90° tilt) and subsequently with its head down (90° tilt). From the day of surgery (day 0), the ICP was recorded for up to 50 days. On the last day, similar physiological manipulation was performed to confirm that the sensor tip still was placed and measuring correctly.

#### Short-term ICP monitoring

Four rats were implanted to monitor ICP for a limited time period to evaluate the feasibility to implant the telemetric device epidurally (Fig. 1c). The measurements were obtained in a daily time-window of 2–5 h for a period of 15 days.

#### Continuous long-term ICP monitoring

**Fig. 2:**
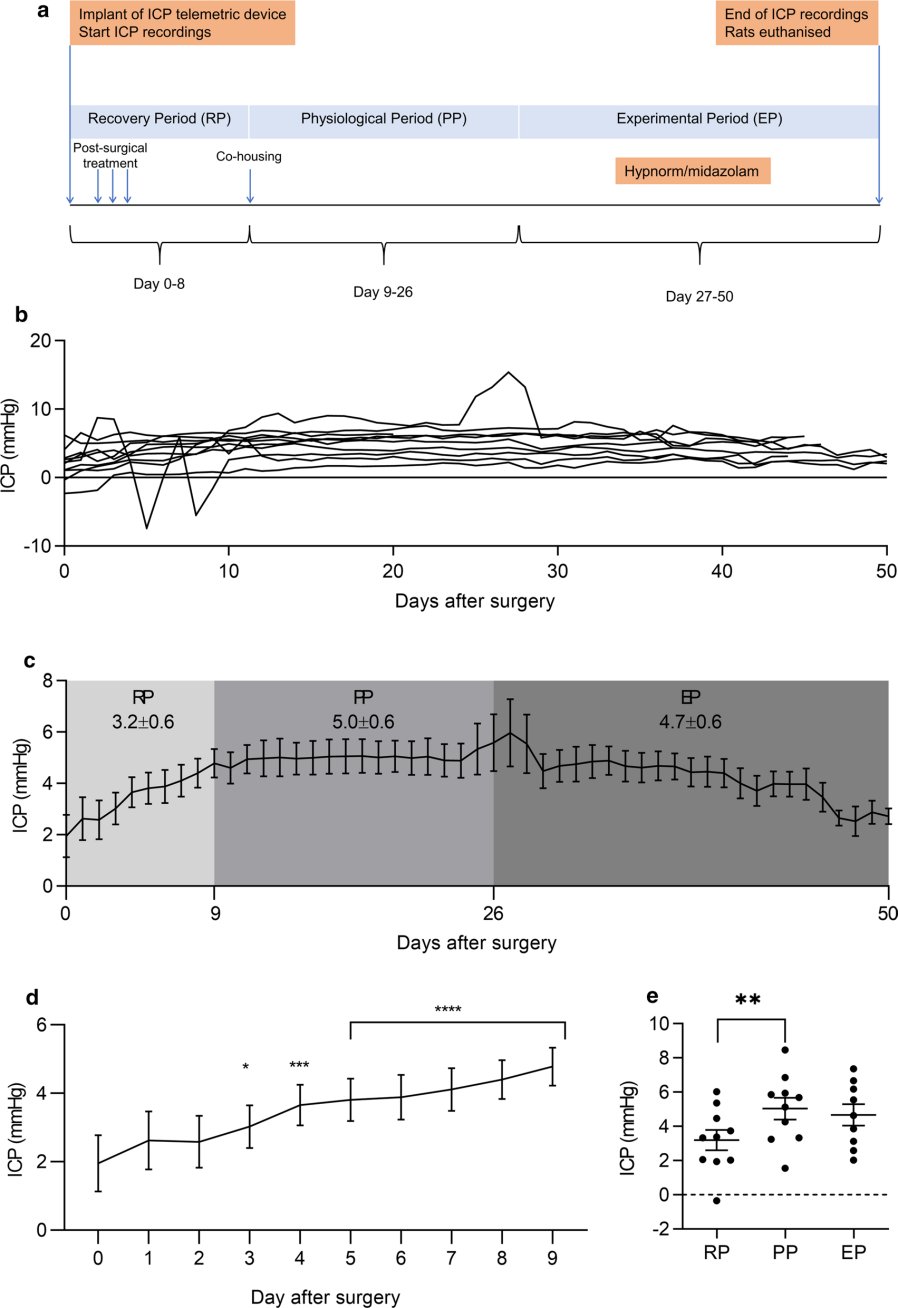
Continuous long-term ICP recording in freely moving rats: **a** The design of the long-term study. The recording period was divided into three periods; recovery period (RP), physiological period (PP) and experimental period (EP). During the RP (day 0–8), the rats were kept single-housed to recover from the surgery. The rats received treatment for 2 days post-surgery (buprenorphine, carprofen, enrofloxacin). When the rats had recovered fully from the surgery, they were co-housed (from approximately day 9). Day 9–26 was considered as the PP, where the rats were not affected by anesthesia or any drugs. During the EP (day 27–50), the anesthetic effect of hypnorm/midazolam was evaluated. **b** Continuous individual daily mean ICP values for each rat over 50 days (n = 10). The ICP measurements were obtained immediately after implantation of the telemetric device on day 0 and continued for up until day 50. **c** Combined daily mean ICP values during RP, PP and EP. **d** Mean ICP values obtained from RP going into the PP (up to 9 days). **e** Repeated measured mixed effects analysis followed by post hoc Tukeys tests for ICP values in the RP, PP and EP. **c**, **d**, **e** Data is presented as mean ± SEM. ** = P<0.01

The second set of animals (n = 10) was implanted with the ICP telemeter for continuous and long-term recording from day 0 up to 50 days. The recording period was divided into three periods (Fig. 2a). The first period was defined as recovery period (RP) from day 0–8 post-surgery. Here the rats were left to recover from the surgery and received post-surgery pharmaceutical treatment for 2 days. The RP was followed by a physiological period (PP) from day 9–26 post-surgery. During this period, the some rats were co-housed, two rats in each cage, when possible (approximately from day 9). The physiological period was followed by an experimental period (EP) from day 27–50 post-surgery. The study design is illustrated in Fig. 2a. The EP was used to test the effect on ICP of a commonly used anesthesia as used for surgeries in this study. Animals received a single dose of 2.7 ml/kg hypnorm/midazolam by s.c. injection.

### Offset testing

The offset testing was done in order to clarify if any sensor tip drift had occurred during the continuous long-term ICP monitoring (Additional file 2: Figure S2). This was performed before and after each use, where the offset or zero levels of the pressure sensors were checked under conditions to replicate use in the animals as performed in a previous study [9]. For each telemetric device, the preimplantation offset value was subtracted from the collected data. One of the implanted telemeters failed and stopped working on day 20. However, this animal was not excluded from the study and ICP data from this animal was analyzed until day 20.

### ICP Spike analysis

To assess the number of ICP spikes, the event count function was used on Lab Chart, where a general sine wave fitting was utilized under default settings and a standard deviation of 8 was used to define an ICP spike. The ICP spike analysis was carried out 3 days (RP) and 10 days (PP) after surgery, using a 3 h period from midday (12:00 p.m.) corresponding to the dark period.

### ICP pulsatility analysis

To assess the ICP pulsatility, the average cyclic height function was obtained by averaging single ICP waves that met the morphological criteria, on LabChart using sinusoidal detection under default settings. Average respiratory pulsatile components were obtained for 3-h time windows from midnight (00:00 a.m.) corresponding to 2.5 h after the start of the light period and from midday (12:00 p.m.) corresponding to 2.5 h into the dark period. These time windows were selected because they were as far from human interaction as possible mitigating unknown and un-investigated effects of human interaction on rat ICP. For waveform analysis, respiration waves which are the slow waves (around 1 Hz) are referred to as, “respiratory waves”. Other slow waves defined as < 0.25 Hz, are referred to as “non-respiratory slow waves”.

### ICP spectrum analysis

The spectral analysis of the ICP waveform was carried out on a 5-min stable section of ICP, i.e. without movement artefacts. Additionally, the fast Fourier transformation (FFT) was carried out on raw, unprocessed 1 kHz data. The inbuilt FFT function of LabChart was utilised where spectral power was obtained utilising the following settings: FFT size of 128 K with a 93.75% window overlap, with a Hann (cosine-bell) data window model. The spectra generated were an average of 23 FFT. For temporal spectrum analysis a stable 5-min baseline spectrum was taken followed by 5-min spectrums taken every 10 min starting at 5 min after hypnorm/midazolam administration.

### In vitro studies

#### Sample preparations

At the end of the study, all animals were euthanized using an overdose of pentobarbital containing lidocaine, the telemeters were explanted, cleaned in an enzymatic detergent, prepared and sterilized for reimplantation. For histological examination, brains from implanted rats (n = 14) and age matched control rats (n = 3) were used. Brains were quickly removed followed by examination of gross histology using a Leica M2125 stereo microscope coupled to a Leica DFC290 camera, brain tissue containing choroid plexus (CP) from the two lateral ventricles was dissected at bregma + 1 mm to bregma − 6 mm. The brain sections were then immersion fixed overnight in 4% paraformaldehyde (PFA) in phosphate buffered saline (PBS) at 4 °C. After fixation, the brain sections were rinsed in increasing concentrations of sucrose in Sörensen’s phosphate buffer, embedded in a gelatin medium (3% gelatin and 30% egg albumin in distilled water) and cryosectioned (12 µm).

#### Immunohistochemical studies

Brain sections from all rats were stained with hematoxylin–eosin (Htx-Eosin) using a standard protocol (Htx 3 min, water rinse, Eosin 1 min) for orientation and evaluation of the implanted brains compared to non-implanted. Immunofluorescence was performed to evaluate the expression of glial fibrillary acidic protein (GFAP). Brain sections were washed for 10 min in PBST (0.25% Triton-X, PBS), prior to blocking in PBS with 5% normal donkey serum for 1 h. Sections were then incubated with buffer (1% BSA, 3% normal donkey serum, PBST) containing mouse anti GFAP antibody (Millipore, IF03L 100 ng/ml) overnight at 4 °C. Following primary antibody incubation, sections were washed 3 × 15 min in PBST prior to incubation with a secondary antibody, Alexa 488 (Invitrogen, A21202, 5 µg/ml) for 1 h. Sections were subsequently washed with PBST for 3 × 15 min. Vectorshield (Vector labs) was used as mounting media. Omission of primary antibody served as negative controls.

#### Microscopic and image analysis

Images were taken on a Nikon Eclipse-Ni epifluorescence microscope coupled to a Nikon Digital Sight DS-U3 camera, where images were acquired using Nis-Elements (4.10.04). Adobe Photoshop v8.0 (Adobe Systems, Mountain View, CA, USA) was used to process for brightness and contrast. To assess the GFAP immunoreactivity, three coronal sections from each brain covering 160 µm were assessed within the area where the sensor tip had been placed. Three regions of interest (ROI) (144 × 144 µm) were selected in each section using ImageJ software (http://rsb.info.nih.gov/ij/). The average intensity was assessed for each ROI, and the mean for that section calculated. For the corpus callosum, ROI was taken at the left, centre and right of the section. The superficial layers of the cortices were assessed, with ROI selected to the left, centre and right of the section. All images were acquired at the same imaging session with the same microscope settings throughout.

### Data and statistical analysis

Data analysis was performed using GraphPad Prism v8.0 software. All data are presented as mean ± standard error of the mean (SEM); n refers to the number of animals. Where n < 7, data were presumed to be normally distributed. Where n ≥ 7, Shapiro–Wilk tests were performed to examine if the data were normally distributed. All data were normally distributed and thus analyzed using parametric tests followed by appropriate post hoc tests as stated in the figure legends. All P values below 5% (P < 0.05) were considered statistically significant; P < 0.05 (*), P < 0.01 (**), P < 0.001 (***) and P < 0.0001 (****).

## Results

### ICP recordings in freely behaving, non-anesthetized rats

#### ICP recordings for 15 days

In the short-term ICP monitoring study, ICP was recorded initially and continued for up to 15 days in four rats. The mean ICP value was 2.9 ± 0.8 mmHg during the 15 days of recording (Fig. 1d). The short-term ICP monitoring study confirmed the validity and feasibility of the telemetric system used to measure ICP.

#### Long-term ICP monitoring for 50 days

In the second group of rats (n = 10), continuous recording for 24 h and long-term ICP monitoring was performed daily from day 0 to day 50 (Fig. 2a). Normal variation in the resting ICP value (day 0) was observed between the rats, ranging from − 2.3 to + 6.2 mmHg with a mean value of 1.9 mmHg (Fig. 2b). The mean ICP values for the three different periods were as following; RP: 3.2 ± 0.6 mmHg, PP: 5.0 ± 0.6 mmHg and EP: 4.7 ± 0.6 mmHg (Fig. 2b, c). In two rats, some short-term fluctuations in the ICP trace were observed (Fig. 2b). For one rat the fluctuations were only observed from day 0 to day 9, and for the other rat the fluctuations were observed only from day 25 to day 28 due to the telemeter failing. These rats had no signs of pain or infection, indicating that the fluctuations were not related to any pathological condition. In general the ICP signals showed a high degree of stability during the whole experimental period of 50 days (Fig. 2b). During the RP, where rats were recovering from surgery, the ICP increased steadily (Fig. 2b–e). In the PP, the ICP values obtained were stable. In the RP, the ICP was significantly lower compared to the PP (P = 0.0035) (Fig. 2e). Therefore, all evaluation of normal ICP physiology was performed during the PP. The mean ICP in EP was not significantly lower than in the PP (P = 0.11). However, given pharmacological testing in this period, the EP was excluded from the physiological analysis.

### ICP and body position

**Fig. 3:**
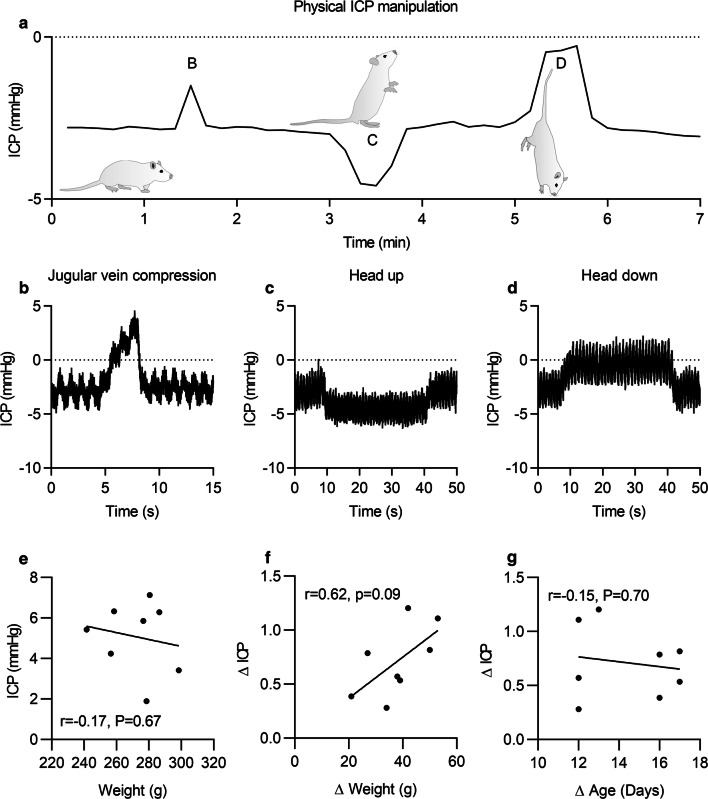
Manipulation of ICP in rodents: **a** Physiological manipulation with change in ICP relative to jugular vein compression followed by postural changes. The increase in ICP during the jugular vein compression (B) is dependent on the force applied. When the rat was kept with its head up the ICP decreased (C) whereas it increased when it was held in a position with its head down (D). **b**–**d** Waveform showing the response in ICP signals to jugular vein compression and response in ICP to different body positions over a period of 15–50 s. The dotted lines represent zero pressure. **e**–**f** Weight and ICP of rats during the physiological period. Scatter graphs detailing the association between ICP and weight (**e**), change in ICP and change in weight (**f**), and change in ICP and change in age (**g**). Pearson correlations, where P < 0.05 was considered significant, n = 8

Physiological manipulation and postural changes were evaluated at day 0 and day 50 while anesthetized (Fig. 3). The signal was assessed by compressing the jugular veins, which gave a significant increase of ICP (Fig. 3a, b). The ICP signal decreased and increased in response to a head up and head down tilt respectively (Fig. 3a, c, d).

### ICP and weight

Given that is has been postulated that body mass can affect ICP, we assessed the association between mass and ICP [17]. No association between absolute mass and daily mean ICP was observed (r = − 0.17, P = 0.67) during the PP (Fig. 3e). However, change in mass showed a weak association with change in ICP (r = 0.62, P = 0.09) (Fig. 3f). This was independent of change in age, where no association was observed (r = − 0.15, P = 0.7) (Fig. 3g).

### ICP wave features and waveform analysis

**Fig. 4:**
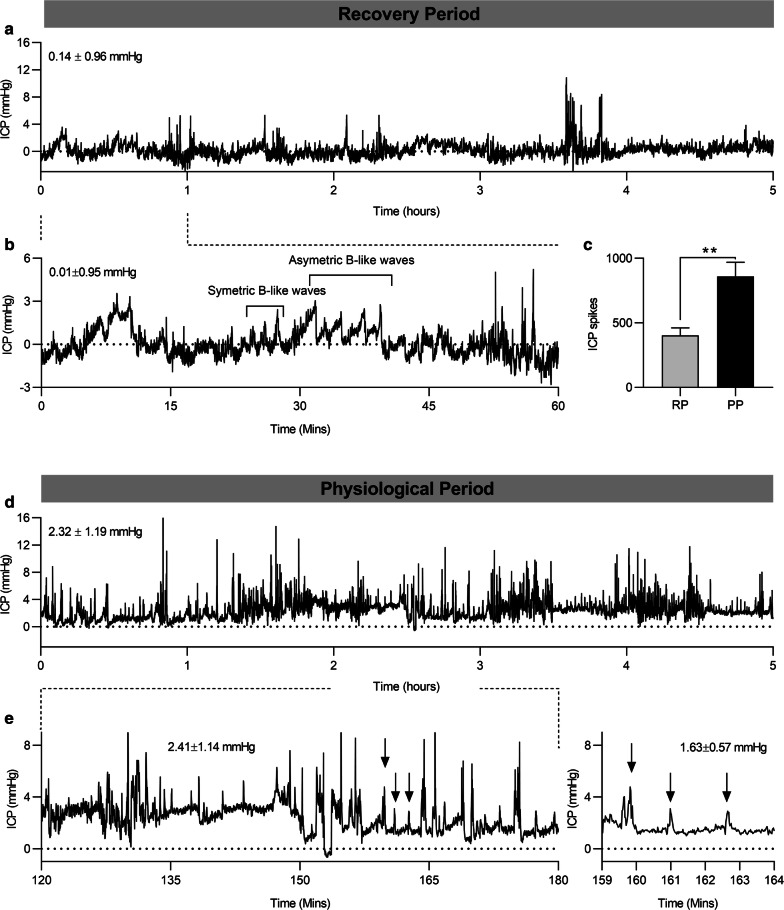
ICP waveform features: Continuous ICP monitoring in rats. **a** Representative ICP trace of a rat during its recovery period during the active phase (dark phase), inserted pressure represents mean ICP for the period shown. **b** Trace detailing ICP waveform features, where B-like ICP waves are highlighted. **c** Bar graph comparing the number of ICP spikes observed in a 3 h window in the RP and PP. **d** Representative ICP trace of a rat in the physiological period during its active phase, inserted pressure represents mean ICP for the period shown. **e** Trace detailing waveform features, where ICP spikes are highlighted with arrows. All traces show 1 s ICP mean values. C presented as mean ± SEM, where data analyzed by paired T-test where N = 10. **P < 0.01

**Fig. 5:**
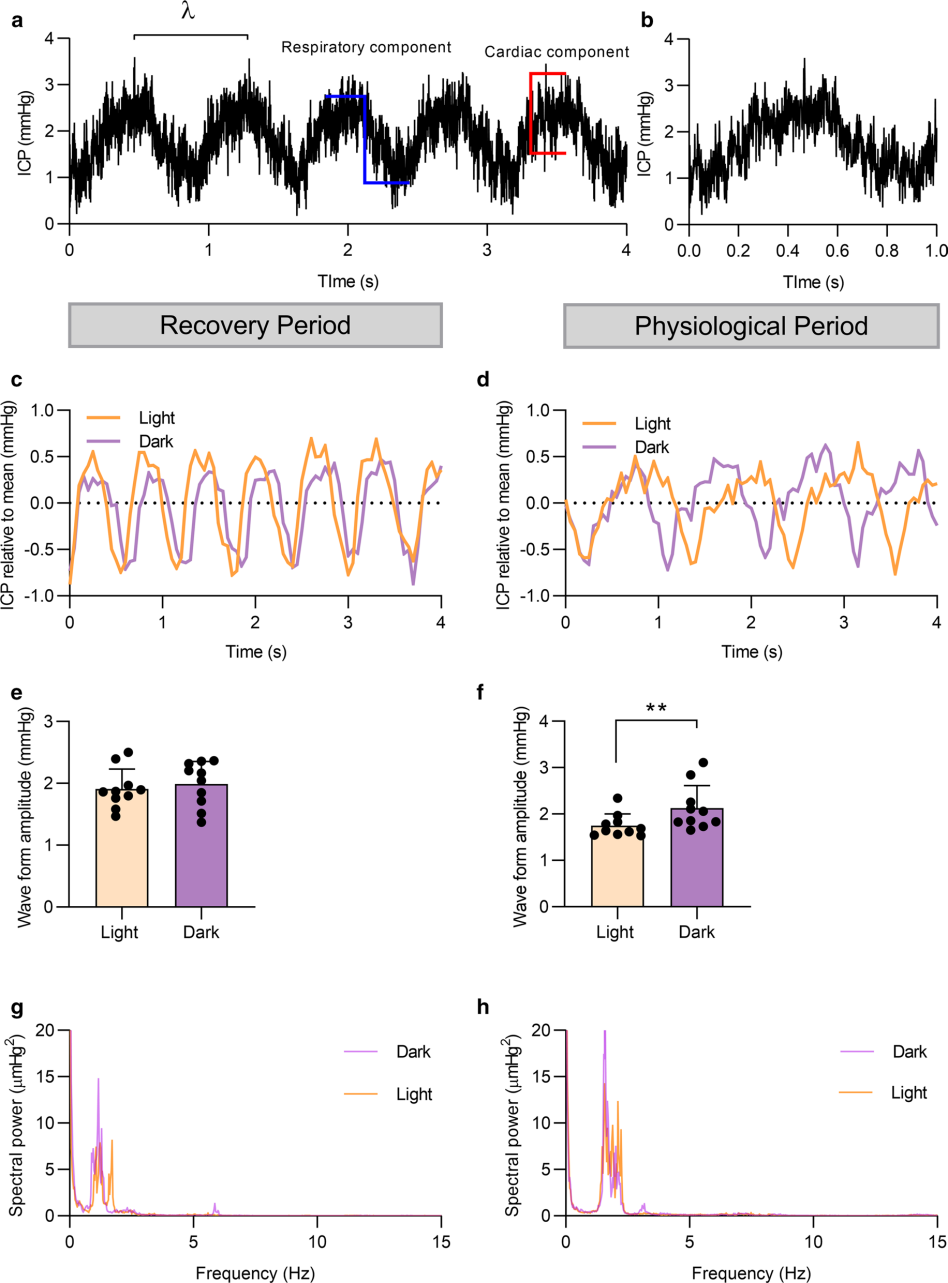
Waveform analysis of ICP measured in freely-moving animals: **a** ICP waveform features. A representative ICP trace detailing ICP waveforms: also showing a respiratory wave (blue) and a cardiac waveform (red), with a 1 kHz sampling frequency. **b** A zoomed in 1 kHz ICP trace detailing one respiratory wavelength. **c**, **d** Representative ICP waveforms displayed at an illustrative 20 Hz sampling mean for the recovery period (RP) and the physiological period (PP) detailing the waveforms in the light (inactive) and dark phase (active). **e**, **f** The ICP waveform amplitude of rats during the light and dark state in the RP and PP showing the increased amplitude in the dark phase of the physiological period. **g** Spectral analysis of the 1 kHz ICP waveform data in both the light and dark phase during the RP and PP (**h**). E–F presented as mean ± SEM, **g**, **h** presented as mean in 0.03 Hz bins. n = 10. Paired t-tests for E and F, **P < 0.01

In the RP active phase, it was demonstrated that there were fewer ICP spikes compared to PP (403.9 ± 56.59 vs 859.2 ± 110.1 spikes, P = 0.0068) (Fig. 4c), perhaps linked to the need to recover from the surgery. ICP waveforms showed similar features to human ICP waveforms, where symmetric B-like waves and asymmetric B-like waves occur over the period of minutes (Fig. 4a, b, d, e). There was a clear sinusoidal shape for the waveform with a respiratory rhythm which was generally 2 mmHg in amplitude (Fig. 5a). Both a respiratory rhythm and a pulsatility rhythm determined by blood pressure were present (Fig. 5b). In the RP there was no difference in the respiratory component of the ICP waveform between the light and dark periods (1.910 ± 0.10 vs 1.991 ± 0.11 mmHg, P = 0.21, (Fig. 5c, e). In contrast, the respiratory component of the PP increased during dark compared to light (1.8 ± 0.1 vs 2.1 ± 0.2 mmHg, P = 0.0087) constituting a 21% increase in in the respiratory component amplitude while active (Fig. 5d, f). To further analyse the ICP waveform, spectral analysis was employed, allowing the generation of a continuous ICP power spectrum for the present system in rats. During the PP, the spectral waveform demonstrated a strong spectral component around 1 Hz, this mirrors frequency of the respiratory waveform, thus this represents the respiratory component (Fig. 5a, g, h). In addition, there was a strong spectral component from 7 mHz to 0.25 Hz, likely representing non-respiratory slow waves. Qualitatively, it was apparent that while rats are recovering from the surgery, they do not exhibit a respiratory waveform around the 1 Hz area (Fig. 5g). Instead this was shifted to the right, indicating an increased respiratory rate while rats recover from the surgery. Of note, the cardiac wave form could not be clearly discriminated in the ICP trace, because on the frequency analysis, there was minimal spectral power in the 5–7 Hz range where the cardiac component would be represented.

### Light and dark variations in ICP

**Fig. 6:**
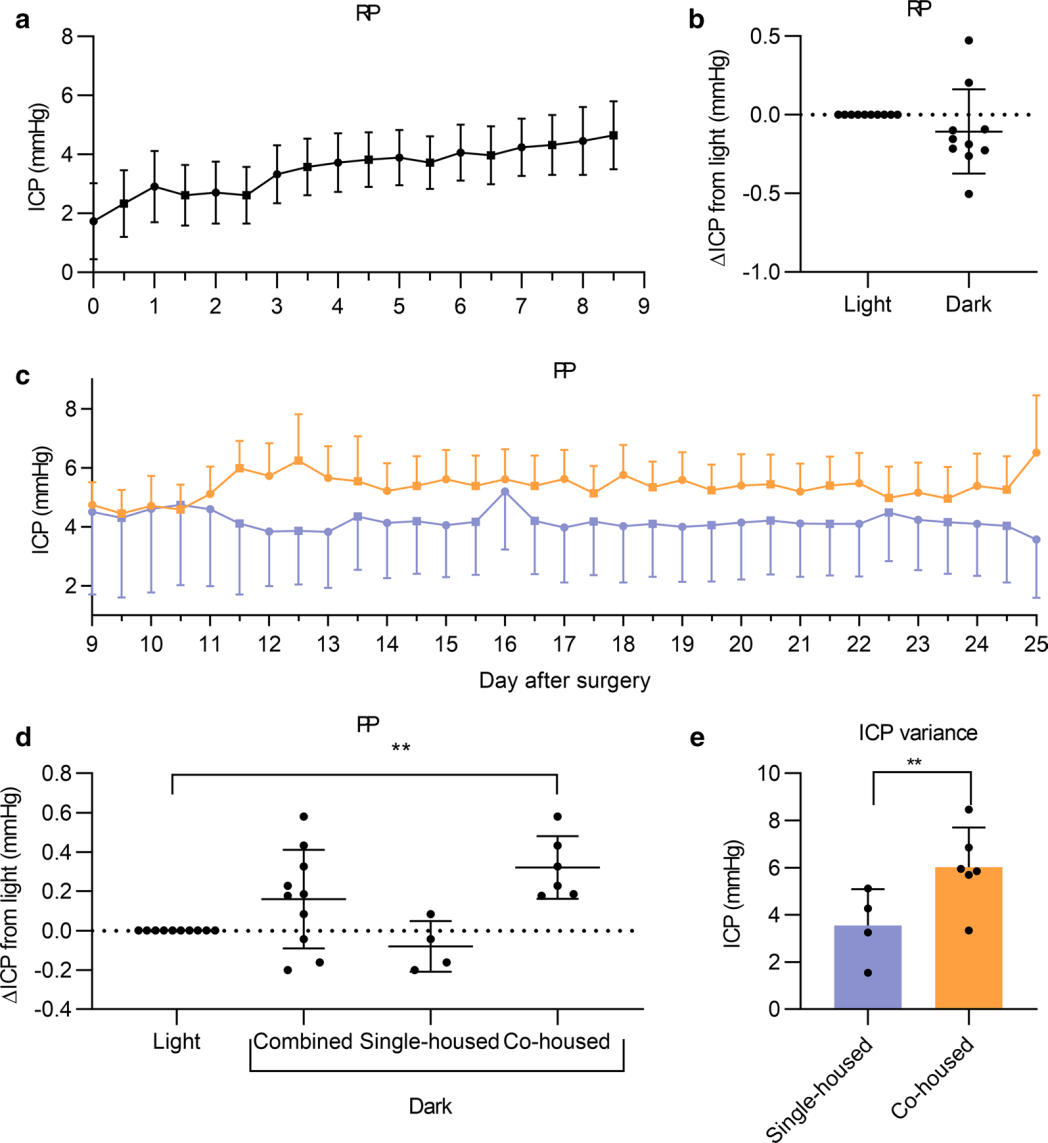
Light and dark variation in ICP: ICP values from continuously measured rats during the recovery period (RP) and physiological period (PP). **a**, **b** Difference in ICP in dark phase compared to light (0.0) in RP, showing the mean ICP for the whole period. **c** Trace detailing light and dark ICP measurements in the PP. In **a** and **c**, circular points represent dark phase values and square points represent light phase values. **d** The difference in ICP during dark phase (active phase) (combined (*n *= 10), single-housed group (n = 4) and co-housed group (*n *= 6)) relative to the ICP in the light phase (inactive phase). The dotted line represents zero change. **E)** Mean ICP in single-housed versus co-housed animals. Paired t-test for B, ANOVA followed by post hoc Dunn’s test for D and unpaired t-test for E. n = 10 and data presented as mean ± SEM

During the RP while animals were single-housed, there was no significant difference between the ICP signals during dark period compared to the light (Δ− 0.1 ± 0.8 mmHg, P = 0.2422) (Fig. 6a, b). In the PP when animals had recovered from the surgery, the rats were divided into two groups: co-housed (n = 6) and single-housed (n = 4). In the co-housed rats there was a significant light–dark difference in ICP (Fig. 6c–e). Here the ICP signal was raised during the dark phase relative to the light phase (Δ0.3 ± 0.1 mmHg, P = 0.0043) (Fig. 6). No difference in light–dark was observed in single-housed rats (Δ-0.1 ± 0.06 mmHg, P = 0.297) (Fig. 6d). The combined group only tend to vary in ICP between light and dark phases (P = 0.0732). Further, we found that the ICP was lower over the whole baseline period in the single-housed animals compared to co-housed animals (3.5 ± 1.5 vs 6.0 ± 1.7 mmHg, P = 0.0457) (Fig. 6c, e).

### Effect of anesthesia on ICP

**Fig. 7:**
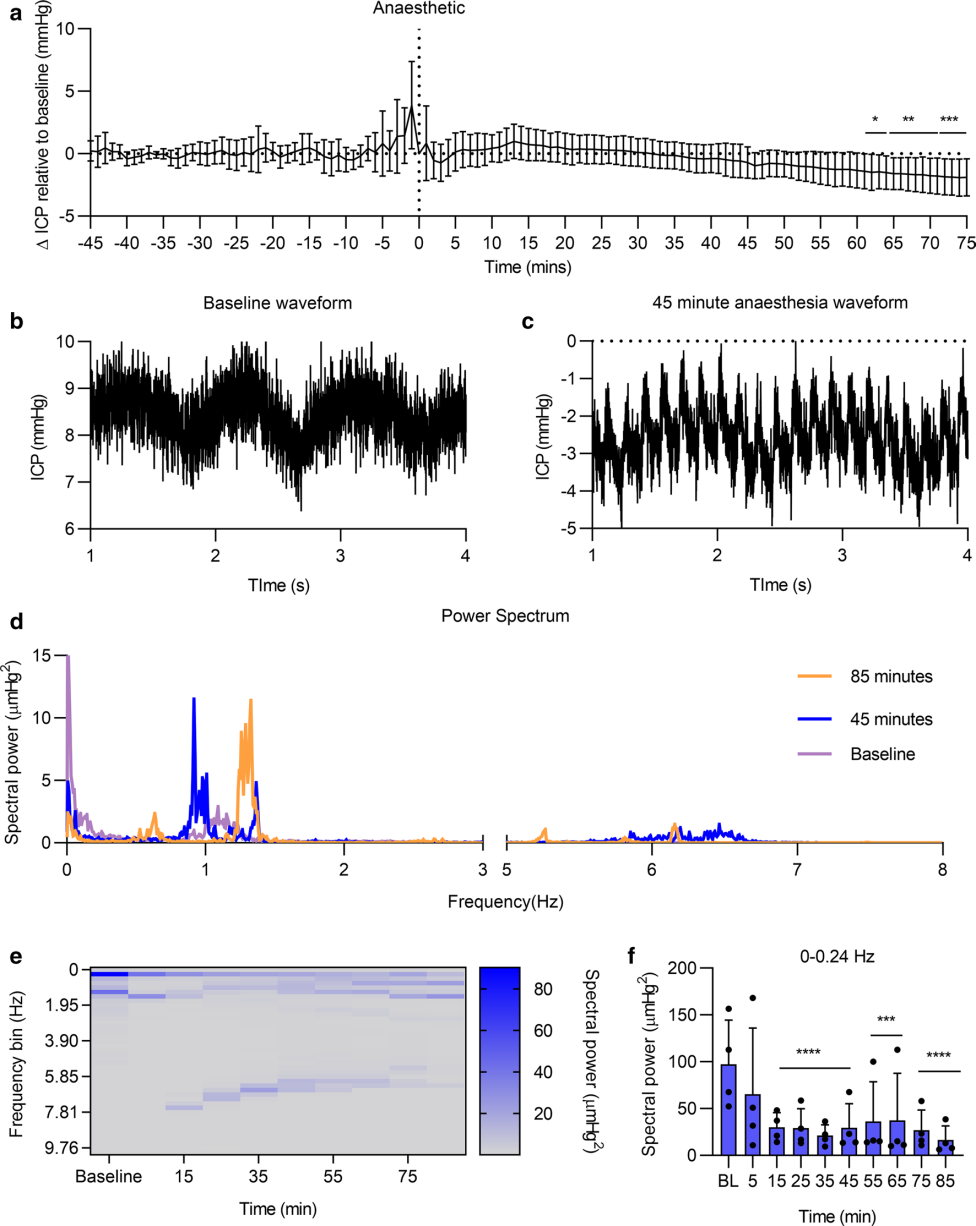
The effects of anesthetic on ICP dynamics: ICP measured in rat receiving hypnorm/midazolam in the experimental phase. **a** ICP trace of ICP before and after the administration of hypnorm/midazolam using 1 min mean values. The peak at time 0 is most likely due to stress from the s.c. injection. Representative ICP waveforms during the baseline period (**b**) and 45 min after anesthesia induction, showing raw 1 kHz data (**c**). **d** A spectrograph detailing the power spectrum of the ICP waveform at baseline, 45 min and 85 min after anesthetic administration. **e** A temporal spectrogram heat map detailing the change in spectral power in frequency bins over time. **f** The effect of hypnorm/midazolam on spectral power in the 0–0.24 Hz frequency bin. Repeated measures ANOVA with post hoc Dunnett’s test for C and D. *** = P < 0.001, **** = P < 0.0001. n = 8 for A, n = 4 for B-D. Data presented as mean ± SEM

The effect of hypnorm/midazolam was evaluated during the EP (Fig. 7). Immediately prior to the injection there was a peak in pressure, due to handling and the stress of injection. After the injection, there was an initial insignificant rise in ICP followed by a continuous decrease in ICP that becomes significant at 61 min (Δ − 1.40 ± 0.49 mmHg, P = 0.03) where pressure continues to decrease at 65 min (Δ − 1.60 ± 0.45 mmHg, P = 0.0075) and at 72 min (Δ − 1.86 ± 0.51 mmHg, P = 0.0007) (Fig. 7a). At 45 min after the induction of anesthesia there was a clear cardiac waveform present on the ICP waveform (Fig. 7c), in contrast to the baseline waveform where cardiac waveform is not clearly visible (Fig. 7b). This is corroborated by spectral analysis of the ICP waveform, whereby a spectral component during anesthesia between 8 and 6 Hz, corresponding to heart rate, was present that was absent during baseline (Fig. 7d, e). Additionally, there was a reduction in spectral power associated with non-respiratory slow ICP waves (0–0.25 Hz) for the duration of the anesthetic period (Fig. 7e).

## In vitro

### Gross histology

Following the experiment, no evidence of trauma or infection was observed in the abdominal cavity or brain (data not shown). Post-mortem examination of the ICP sensor tip position showed that they were lying on top of the dura mater and cortex, none of the sensor tips were angled down into the cortical tissue. In all implanted rats, the cranial bones were not penetrated by the drill holes or the anchoring screws.

### Immunohistochemical studies

**Fig. 8:**
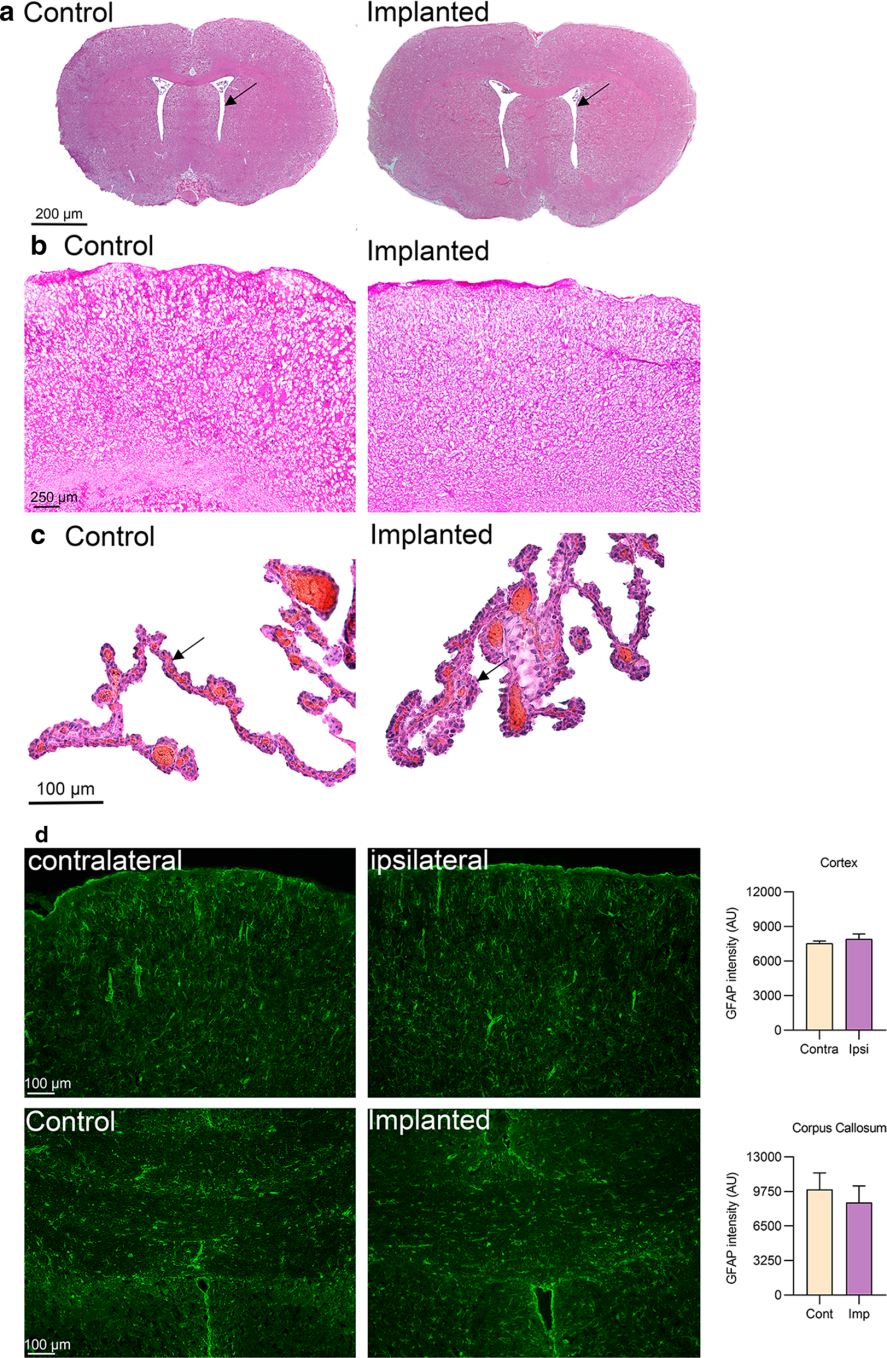
Histological examination of brains from implanted animals: The areas of interest were cerebral cortex were the sensor tip approximately had been placed. **a** Whole brain sections from control (non-implanted) and animals implanted with the telemetric device for 50 days (implanted). Ventricular morphology was similar in control and implanted rats, showing no signs of ventriculomegaly and hydrocephalus in the implanted animals (arrows). **b** The cerebral cortex at higher magnification from control (non-implanted) and implanted brains for 50 days. The tissues from implanted rats were intact with no signs of tissue damage or bleeding when compared to tissue from non-implanted rats. **c** Histological examination of choroid plexus (CP). The morphology of the CP structures was similar in tissue from implanted rats for 50 days (implanted) compared to tissue from non-implanted rats (control). The morphology of CP was normal in all animals; the ependymal cells lining the ventricles with the apical side of the membrane facing the lumen of the ventricle was clearly defined as well as the basolateral side of the membrane facing the blood capillaries (arrows). **d** Assessment of GFAP staining in implanted and non-implanted brains. Upper panel shows representative micrographs of the contralateral and ipsilateral cortices of implanted brains with graph of GFAP intensity. Lower panel shows representative micrographs of corpus callosum of implanted and non-implanted (control) brains and graph with GFAP intensity in cortex. n = 3 control, n = 8 implanted. t-test for corpus callosum and paired t-test for cortical intensity measurement. Data presented as mean ± SEM

Htx-Eosin-staining revealed no evidence of cerebral damage in posterior cortical areas (close to where the sensor tip was placed) in implanted rats compared to controls (Fig. 8a, b). Ventricular morphology was similar in control and implanted rats, providing no evidence of ventriculomegaly and hydrocephalus (Fig. 8a). It was also clear that CP in all animals showed normal morphology (Fig. 8c). Further, no difference in GFAP immunoreactivity was detected between the ipsilateral and contralateral cortical hemispheres (7533 ± 196.0 vs 7913 ± 442.2 AU, P = 0.29) (Fig. 8d). Additionally, we assessed corpus callosum GFAP immunoreactivity to determine if the implant was causing a more systemic gliosis, where no difference in the GFAP immunoreactivity between non-implanted and animals implanted for 50 days was observed (9970 ± 890.6 vs 8739 ± 539.9 AU, P = 0.26) (Fig. 8d).

## Discussion

Valid and long-term ICP monitoring in the preclinical setting is of crucial importance for understanding ICP regulation. In line with this goal, we used a novel, state-of-the-art system that enables continuous ICP measurements in freely-moving rats. We investigated how the dynamics and ICP physiology are altered under different physiological states for up to 50 days of ICP monitoring.

### Long term ICP features and effects of surgery/recovery

This study demonstrated chronic, continuous ICP monitoring in freely-moving rats for up to 50 days via a telemetric device. To our knowledge, this is the longest recording time for ICP monitoring in conscious rats. The different physiological parameters evaluated in the current study were performed in female rats. Another study using the same telemeter system in male rats showed similar ICP mean values [9]. Furthermore, we demonstrated earlier that there is no difference in the mean resting ICP and amplitude between female and male rats [8]. In the current study, the long-term recording period was divided into three periods; RP (day 0–8), PP (day 9–26) and EP (day 27–50) with focus on evaluation of the ICP signal and the associated physiology throughout the different periods. We found that the resting ICP value for the rats at the day of surgery increased on the following days. Clear respiratory rhythm and a pulsatility determined by blood pressure were present on the ICP signal. However, clear cardiac oscillation spectral components were not present, or were minimal and were not a major component of the ICP traces either following surgery or after they had recovered. When ICP is pathologically increased in hydrocephalic rats, then the cardiac oscillation is present on the ICP signal, suggesting that physiological cerebral compliance does not allow resolution of blood pressure on ICP in rats [9]. However, we did observe cardiac oscillation when the animals were under anaesthesia, suggesting that cerebral compliance is altered by anaesthetic.

There was a significant difference between the mean ICP values obtained from the different recording periods, where the mean ICP in the RP was lower than the mean ICP in the PP. These results suggest the rat ICP is affected by the surgical procedure, including the use of anesthesia and post-surgery treatments. We also demonstrated that in the PP during the dark period (active phase of the animals) there are both ICP spikes and increased baseline ICP. However, in the RP active phase, we demonstrated a reduced number of ICP spikes, perhaps linked to the need to recover from the surgery and reduced activity.

In the clinical setting, B waves have been described [18]. The classical B waves are defined as short repeating elevations in ICP with a frequency of 0.5–2 waves/min reflecting vasogenic activity of cerebral autoregulation [19, 20]. Subsequently, B waves are further divided into symmetrical and asymmetrical B waves [20]. While B waves had originally been considered a pathologic phenomenon, they have been observed in healthy individuals [19, 21–24]. Interestingly, we observed ICP waveforms that show similarity to human ICP waveforms where both symmetric and asymmetric B-like waves were observed and they both occurred over the order of minutes during the RP analogous to the human B waves. This likely demonstrates dysregulated ICP compliance and homeostasis directly following the surgery. Thereby, it is of great importance to allow the rats to recover from these kinds of procedures in order to obtain valid physiological data such as ICP.

When we compared single-housed animals to co-housed animals, the mean ICP was lower in the single-housed animals during the PP. This difference may be due to the fact that single-housed animals are more stressed, as it is known that housing conditions affects their physiology and that single-housed animals have increased stress levels [25]. In support of this it has been demonstrated that social instability leads to reduced weight gain, induced thymus involution, adrenal hypertrophy and elevated plasma corticosterone levels in female rats [26, 27]. Co-housing female rats tends to diminish stress [26]. Our results suggest that disturbed social interaction (single-housing) in female rats influences ICP physiology.

### ICP is affected by body positions

Rats predominantly live in the prone position, and the cranium is not at the top of a fluid column but level with the rest of the CSF fluid compartment. Therefore, movement and flexion of the spine and cranial orientation is likely to have a large effect on the ICP. Interestingly, we demonstrated that different body positions of the animals affected the ICP. During conditions where a rat’s head was held down (90° tilt), there was an immediate increase in ICP and the ICP decreased in response to holding the rat with its head up (90° tilt). Holding the rat with its head upwards is analogous the human spinal orientation when standing. In support, it has been shown that ICP also changes in humans during different body postures where switching from a supine position to an upright position causes a decrease in ICP [15, 16, 28]. In line with this, we were able to mimic the effect of human body positions on ICP in rats by altering the position/orientation of the animal. In humans, at the horizontal position, ICP reference values are estimated to be 7–15 mmHg, however these values can be lower and negative and should be considered to be normal (supine position, mean ICP = 0.5 mmHg; vertical position, mean ICP = − 3.7 mmHg) [16].

### The impact of body weight on ICP

Obesity has been suggested to impair CSF absorption pathways and is a predominant phenotype shared by the large majority of patients suffering from IIH [29, 30]. Furthermore, we previously demonstrated that genetically obese rats have higher ICP compared to lean rats, suggesting that obesity may influence ICP [31]. In the current study, we found that with normal weight gain during the PP (17 days) there was a trend to increase in ICP. This was independent of age, supporting the idea that obesity has an impact on ICP. Further studies with more animals, longer weight gain period and rapid weight gain are needed to evaluate this further.

### Light and dark variations in ICP

We found that co-housed rats had a significantly higher ICP during the dark when compared to light phase. Interestingly, this variation was not observed in the single-housed rats. This may again be due to that the rats are more stressed when housed alone and that this might affect their natural behavior and diurnal variation [25, 32]. Furthermore, we demonstrate that while animals were recovering from surgery there was no difference in the respiratory component amplitude of the ICP waveform between the light and dark periods. In contrast, the respiratory component amplitude in the PP was increased by 21% during the dark compared to the light period. This adds further evidence that in order to obtain normal ICP physiology, rats must be fully recovered from the surgery. Diurnal variation in ICP has previously been studied in both basic research and in clinical studies. In basic research, the data is inconsistent. In an older study, ICP was measured from the lateral ventricle in relatively unrestrained rats for a period of 24 h using a fluid-filled system [33]. A significant higher ICP during dark phase was noted when compared to light phase which is in accordance with our results [33]. In contrast to this, two other groups did not find any ICP variation between the light–dark phases [9, 34]. However, in these studies, the light–dark ICP variation was investigated in single-housed rats and the ICP was recorded over a short period of time. Furthermore, light–dark variations were measured after a relatively short time post-surgery (24 h–7 days). We also observed no light–dark differences directly after surgery up to 8 days in single-housed animals. This again indicates the importance of performing such physiological measurements under conditions where animals are fully recovered and in their normal state.

In clinical human studies, higher ICP has been reported during night time when compared to day time [35]. The patients included were considered as normal as possible, as measuring ICP in healthy subjects without symptoms of ICP pathology is unethical. Therefore, it is unknown if similar ICP values would be obtained in completely healthy persons with normal CSF physiology. Also, the day–night ICP variation could to be due to the hydrostatic increase in ICP from lying down in a horizontal position when sleeping. Furthermore, sleep may cause changes in physiological processes such as CSF reabsorption and metabolism (activation of the glymphatic system) affecting ICP dynamics [36]. In this study we were unable to monitor locomotor activity of the animals, and consequently cannot endorse whether these differences are due to sleep/circadian rhythm or higher locomotor activity during the dark period.

### Effect of anesthesia on ICP

Previous studies monitoring ICP in animals have utilized anesthetics, as necessitated by the experimental animals being tethered. Given this, we assessed the effects of a commonly used anesthetic on ICP dynamics. We demonstrated that hypnorm/midazolam-induced anesthesia has multimodal effects on ICP. There was a trend towards an increase in ICP during the first 15 min after anesthesia administration followed by a significant decrease in ICP. The decrease in ICP may explain a generally lower ICP directly after surgery on day 0 as compared to the following days. These results are comparable with a previous study showing a lower CSF pressure in anesthetized rats compared to non-anesthetized rats [7]. Spectral analysis of the ICP waveform demonstrated several differential features after administration of the anesthetic agent. A reduction in spectral power associated with non-respiratory slow waves (0–0.25 Hz) for the duration of the anesthetic period was seen in the present study. Additionally, we observed spectral power in the frequency 5–7 Hz appearing during anesthesia, which was absent when the animals were awake. This is likely the heart rate of the rats which is around 5.4 Hz [37]. It remains to be determined whether the observed effects are derived from a combination of these agents, or which of the agents is causing the observed ICP changes. These results clearly demonstrate that anesthesia in rodents affects ICP and potentially alters the cerebral compliance. Taken together, these results poise the discussion that previous approaches to measure ICP in anesthetized rats might present compromised ICP values which were affected by the anesthesia. Anesthetic agents may affect the dynamics of the cerebral vasculature through direct effects on the vessels as well as modulation of the regulator mechanism [38]. Anesthetic change in ICP as observed in the current study could be caused by a direct effect on the vasculature and the cerebral blood flow (CBF). Animal studies have shown that hypnorm caused a decrease in CBF during several hours. In patients, intravenous anesthetics such as propofol, thiopental and etomidate reduced CBF and ICP [39]. In TBI patients undergoing surgery, there was significant reduction in ICP in patients receiving propofol compared to those who received isoflurane [40]. Isoflurane and sevoflurane used in the clinical setting have shown increase in ICP [41–44]. In contrast, other studies demonstrated that ICP remained unchanged during the use of isoflurane or sevoflurane [45–47]. The effect of anesthetic agents in patients strongly depends on the type of the brain injury, ventilation and in combination with other sedative agents. Therefore, it is of great value to evaluate the effect of anesthetic agents in a controlled environment such as the preclinical setting.

### Well-being and pathology assessment

The animals recovered well from the surgery as indicated from their weight recovery, water consumption and resumption of normal behavior. Since transferring rodents from cage to cage is a stressor, we exclusively measured ICP in the rat’s home cage, thus reducing the potential effects of stress on ICP [48]. To our knowledge, we are the first group to measure ICP from the epidural space using this type of telemetric device. Previously, this telemetric system has been used in few studies, where ICP was measured from the subdural space or placed intraventricularly using male rats [9, 13, 14]. Both subdural and intraventricular measurements are more invasive procedures associated with a higher risk of infection, symptoms of neurological malfunction and hydrocephalus [8, 49]. Previously, Uldall et al. compared two recording sites, the lateral ventricle and the epidural space, with no difference in recorded ICP values [8]. Therefore, in this study where we aimed for long-term survival time and stability, ICP recordings from the epidural space was performed. Histological examination demonstrated that the sensor tip did not cause tissue damage, inflammation or bleeding. Importantly, the ventricles were of normal size in all rats, revealing no signs of ventricular enlargement. This does not exclude potential damage to the dura as this was not assessed. Furthermore, the structure of CP was not changed in implanted rats. GFAP immunoreactivity demonstrated that the catheter does not cause astrogliosis over the 50 day of implantation either in superficial grey matter areas or deep white matter areas. Altogether, this supports that animals remained healthy during the entire time of measurements and that animals were housed under conditions ensuring their physiological and behavioral needs.

### Limitations of the study

In this study, ICP recordings were performed from the epidural space as we aimed for long-term survival time in the animals and other recording sites are more invasive [5]. This is of great importance in the development of animal models for disorders such as IIH where ICP homeostasis is perturbed without cerebral trauma. In the preclinical setting, rodent ICP values have been reported to vary between 2 and 8 mmHg with a mean value around 6 mmHg [5]. In this long-term study, the mean values in the different periods were in the same range as previous studies using other ICP recording sites (RP: 3.2 mmHg, PP: 5.0 mmHg and EP: 4.6 mmHg). Further, our previous studies have also confirmed that monitoring ICP from the epidural site gives a robust increase in ICP in a hydrocephalus rat model, ICP value of > 10 mmHg, as observed with other ICP monitoring sites [50]. Further, we have shown clear ICP responses to drugs that are believed/known to lower ICP using the epidural site for ICP recording [50–52]. As described earlier, in rodents, there is no difference in ICP measurements from the lateral ventricle or the epidural space [8]. Monitoring from the epidural site is preferred in animal studies as it is less invasive and safe for the animals. Also in the clinic, epidural ICP monitoring is advantageous by being less invasive, with less risk for complications compared to parenchymal sensor placement. However, ICP monitoring from the epidural space is not common in the clinic as studies have reported errors in mean ICP monitoring from the epidural site producing artificially high values [53–55]. This may be due to the specific characteristics of the epidural intracranial space in humans or the epidural sensors not having enough accuracy for clinical use. Thereby, there seems to be underlying differences between the human and rodent ICP recording sites and the obtained data in this study might not be directly translated to human ICP physiology. The current study was able to confirm and replicate some of the human ICP physiology parameters such as change in ICP with body position, day–night variations and anesthetic effects. Although, few centers have although reported the clinical use of epidural ICP [53, 56, 57]. It has been suggested that epidural ICP monitoring in humans can still provide the pulsatile ICP properly which could still be beneficial for certain patients [53]. In a clinical study, comparing ICP sensors placed within the brain parenchyma and the epidural space demonstrated that the two sites showed differences in mean pressure. The study suggested however that epidural ICP monitoring would be useful for determining ICP waveform parameters [57]. Therefore, in the clinical setting, the limitations with epidural ICP measurements are related to static ICP measurements but not to measurements of the ICP waveform.

According to the manufacture, Kaha Sciences, the maximum drift of the pressure sensor is 4 mmHg per month. In this study the sensors drifted less than that, however for ICP measurements a small drift in the signal could provide a substantial error in the recording values. We observed that in the individual animals the ICP signal was very stable, giving us confidence that the obtained values were not affected by drift. Since it cannot be determined when this minimal drift started, we corrected for this by subtracting the preimplantation offset for each sensor in each rat from the collected data. Another limitation with the current study is that we were not able to link movement of the animals to changes in ICP while animals were freely moving in their home cages. Effect of body positions were evaluated while animals were under anesthesia and similar changes were found while observing freely moving animals. However, in the future, parallel recording of the animals’ movement and ICP while they are in their home cage will provide valuable data in order to understand normal physiologic body pressure tonus in rodents.

## Conclusions

This study provides unprecedented insight into ICP in freely moving rats while circumventing limitations of previous studies regarding anesthetic effects, short term recording versus long term recording as well as recording area. For the first time, this study demonstrates continuous ICP monitoring in freely-moving and co-housed rats for up to 50 days with a high degree of stability. We also demonstrate that using the epidural space in rodents is safe and provides stable ICP data. The study reveals that under normal physiological conditions, ICP dynamics and its waveforms in rodents are influenced by body positions, recovery from surgery, use of anesthesia, weight, light–dark difference and housing conditions. The study demonstrates also the great importance of performing ICP measurement in freely-moving animals housed under normal conditions. Further, we show that co-housed rats showed higher ICP during dark when compared to light phase. Surgery, single-housing and the use of anesthesia decreased ICP and affected its waveforms. Therefore, all these parameters must be taken into considerations in future studies designed to develop animal models for ICP-related disorders and exploring drug targets for these disorders. Our ability to record ICP in conscious rats living in their home cage opens up opportunities for future studies to investigate how the dynamics and mean level of ICP may change under disease development in conditions with impaired ICP homeostasis.

## Supplementary information

**Additional file 1: Figure S1.** Weight and water consumption after surgery. A) Weight and water consumption during the recovery period (RP). B) Weight monitoring during RP and the physiological period (PP). Data is presented as the percentage mean daily weight ± SEM and mean daily water consumption ± SEM. n = 14.

**Additional file 2: Figure S2.** Offset testing of sensor drift. Graph illustrating the sensor drift that has occurred in each of the telemetric devices during implantation time in the continuous long-term ICP monitoring study. The offset testing was done according to the manufacturer’s instruction. The mean absolute value of the offsets was 0.1 ± 0.8 mmHg prior to implantation and 1.9 ± 0.9 mmHg after implantation and the mean absolute difference between the offset before implantation and after explanation was 2.1 mmHg over 50 days of implantation.

## Data Availability

All data generated or analysed during this study are included in this published article [and its additional information files].

## Abbreviations

CSF: Cerebrospinal fluid
CP: Choroid plexus
CPe: CP epithelial
EP: Experimental period
FFT: Fast fourier transformation
GFAP: Glial fibrillary acidic protein
IIH: Idiopathic intracranial hypertension
ICP: Intracranial pressure
RP: Recovery period
PP: Physiological period
s.c.: Subcutaneous injection
TBI: Traumatic brain injury
