# Supplementary figures and images for: Long-term monitoring of intracranial pressure in freely-moving rats; impact of different physiological states
Source: Fluids Barriers CNS. 2020 Jun 9;17:39. doi: 10.1186/s12987-020-00199-z (PMC7285467; doi:10.1186/s12987-020-00199-z)

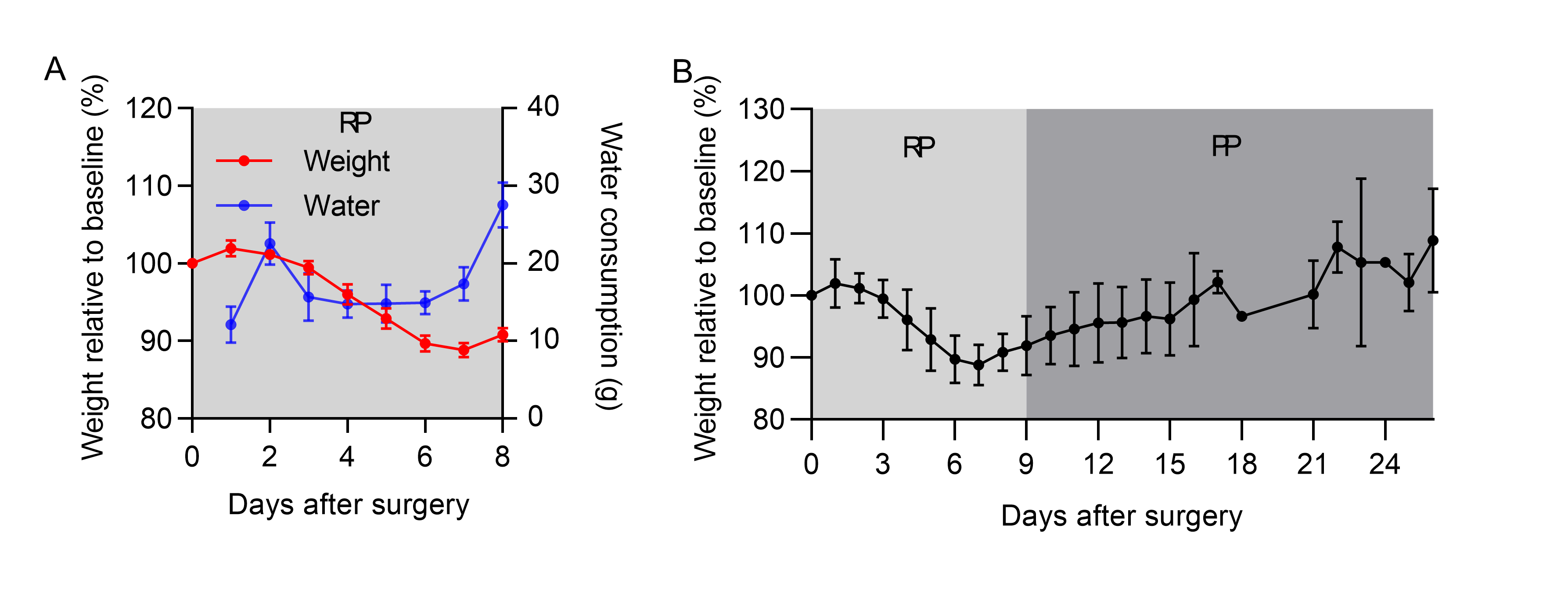

Supplement: Supplementary file 1 — Additional file 1: Figure S1. Weight and water consumption after surgery. A) Weight and water consumption during the recovery period (RP). B) Weight monitoring during RP and the physiological period (PP). Data is presented as the percentage mean daily weight ± SEM and mean daily water consumption ± SEM. n = 14. [file 12987_2020_199_MOESM1_ESM.tif]

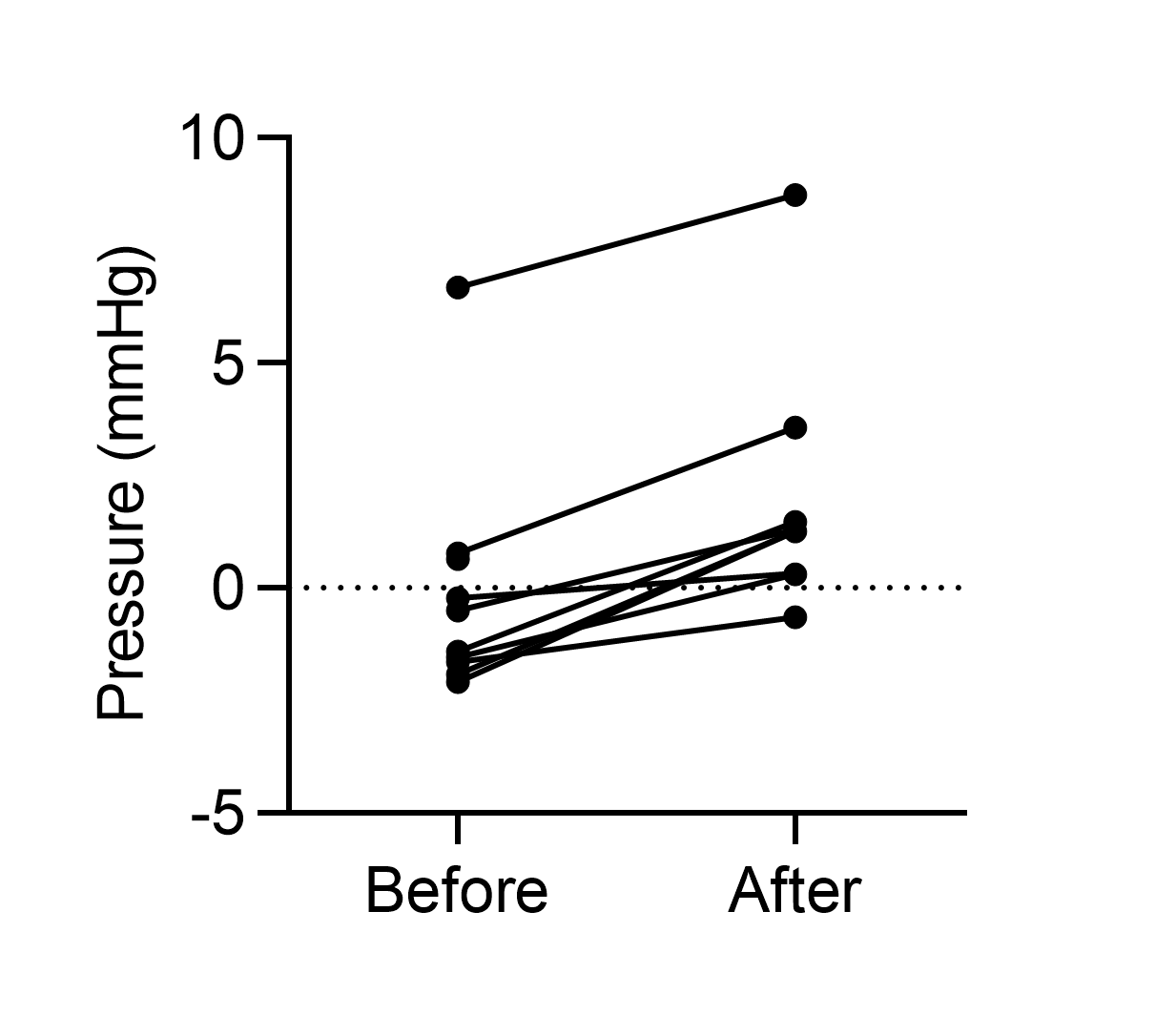

Supplement: Supplementary file 2 — Additional file 2: Figure S2. Offset testing of sensor drift. Graph illustrating the sensor drift that has occurred in each of the telemetric devices during implantation time in the continuous long-term ICP monitoring study. The offset testing was done according to the manufacturer’s instruction. The mean absolute value of the offsets was 0.1 ± 0.8 mmHg prior to implantation and 1.9 ± 0.9 mmHg after implantation and the mean absolute difference between the offset before implantation and after explanation was 2.1 mmHg over 50 days of implantation. [file 12987_2020_199_MOESM2_ESM.tif]
